# Deviating dental arch morphology in mild coronal craniosynostosis syndromes

**DOI:** 10.1007/s00784-018-2710-9

**Published:** 2018-11-03

**Authors:** T. M. Choi, L. Kragt, J. A. C. Goos, I. M. J. Mathijssen, E. B. Wolvius, E. M. Ongkosuwito

**Affiliations:** 1 Department of Oral Maxillofacial Surgery, Special Dental Care and Orthodontics, Dutch Craniofacial Center, Erasmus University Medical Center, Wytemaweg 80, 3015 CN Rotterdam, the Netherlands; 2 Department of Orthodontics and Craniofacial Biology, Radboud University Medical Center, Philips van Leydenlaan 25, 6525 EX Nijmegen, the Netherlands; 3 Department of Plastic and Reconstructive Surgery and Hand Surgery, Dutch Craniofacial Center, Erasmus University Medical Center, Wytemaweg 80, 3015 CN Rotterdam, the Netherlands

**Keywords:** Dental arch dimensions, Jaw relationship, Syndromic craniosynostosis, Craniofacial anomalies, Craniofacial biology/genetics, Growth/development, Jaw biomechanics, Tooth development, Orthodontic(s)

## Abstract

**Objectives:**

To determine whether the intramaxillary relationship of patients with Muenke syndrome and Saethre-Chotzen syndrome or *TCF12*-related craniosynostosis are systematically different than those of a control group.

**Material and methods:**

Forty-eight patients (34 patients with Muenke syndrome, 8 patients with Saethre-Chotzen syndrome, and 6 patients with *TCF12*-related craniosynostosis) born between 1982 and 2010 (age range 4.84 to 16.83 years) that were treated at the Department of Oral Maxillofacial Surgery, Special Dental Care and Orthodontics, Children’s Hospital Erasmus University Medical Center, Sophia, Rotterdam, the Netherlands, were included. Forty-seven syndromic patients had undergone one craniofacial surgery according to the craniofacial team protocol. The dental arch measurements intercanine width (ICW), intermolar width (IMW), arch depth (AD), and arch length (AL) were calculated. The control group existed of 329 nonsyndromic children.

**Results:**

All dental arch dimensions in Muenke (ICW, IMW, AL, *p* < 0.001, ADmax, *p* = 0.008; ADman, *p* = 0.002), Saethre-Chotzen syndrome, or *TCF12*-related craniosynostosis patients (ICWmax, *p* = 0.005; ICWman, IMWmax, AL, *p* < 0.001) were statistically significantly smaller than those of the control group.

**Conclusions:**

In this study, we showed that the dental arches of the maxilla and the mandible of patients with Muenke syndrome and Saethre-Chotzen syndrome or *TCF12*-related craniosynostosis are smaller compared to those of a control group.

**Clinical relevance:**

To gain better understanding of the sutural involvement in the midface and support treatment capabilities of medical and dental specialists in these patients, we suggest the concentration of patients with Muenke and Saethre-Chotzen syndromes or *TCF12*-related craniosynostosis in specialized teams for a multi-disciplinary approach and treatment.

## Introduction

Syndromic craniosynostosis is characterized by the premature fusion of one or more cranial sutures and other craniofacial deformities at birth. Syndromic craniosynostosis shows a wide spectrum of phenotypes. For instance, maxillary hypoplasia is commonly reported in the severe craniosynostosis syndromes, such as the Apert and Crouzon syndrome [1]. As a result, children with the Apert and Crouzon syndrome have smaller dental arch dimensions compared to those of healthy Dutch children [2–4]. To our knowledge, the dental arch dimensions in Muenke and Saethre-Chotzen syndromes or *TCF12*-related craniosynostosis have not been assessed nor is maxillary hypoplasia commonly reported in these three craniosynostosis syndromes [5–8].

The reported phenotype of these three craniosynostosis syndromes is quite similar [9–12]. These three craniosynostosis syndromes can all be genetically confirmed. Muenke syndrome is caused by the P250R mutation in *FGFR3* [9]. Saethre-Chotzen syndrome is caused by mutations in or re-arrangements in *TWIST1* [13] and *TCF12*-related craniosynostosis is caused by mutations or re-arrangements in *TCF12* [11–14]*.* Saethre-Chotzen syndrome or *TCF12*-related craniosynostosis present a genetic close relationship and most of the phenotypic features related to *TCF12*-related craniosynostosis mutations or re-arrangements resemble those of Saethre-Chotzen syndrome with a large clinical spectrum [15]. This suggests that Saethre-Chotzen syndrome or *TCF12*-related craniosynostosis may share other craniofacial features, such as the size of the maxilla or the dental arch dimensions. Based on the craniofacial knowledge in the Apert and Crouzon syndrome, we expect that similar craniofacial characteristics should be present in a lesser extent in Muenke syndrome and Saethre-Chotzen syndrome or *TCF12*-related craniosynostosis*.*

The aim of our study is to compare the dental arch dimensions of patients with Muenke syndrome and Saethre-Chotzen syndrome or *TCF12*-related craniosynostosis patients to those of a control group of healthy Dutch children, to investigate whether their jaw sizes are systematically smaller and whether the intermaxillary relationships of patients with Muenke and Saethre-Chotzen syndromes or *TCF12*-related craniosynostosis differ compared to those of the Dutch controls.

## Material and methods

Patient records were collected as part of orthodontic record taking according to the treatment protocol used by the craniofacial team. Subsequently, a search was conducted for the available dental casts in Digimodel 2.3.7 (OrthoProof B.V, the Netherlands) where dental casts were saved. The dental casts were transformed into digital models (OrthoProof, Nieuwegein, the Netherlands). Impressions and wax bites were scanned using a Flash CT scanner (model FCT-1600; Hytec, Los Alamos, NM, USA) at 160 kV with a voxel resolution of 0.05 mm.

### Study population

**Fig. 1:**
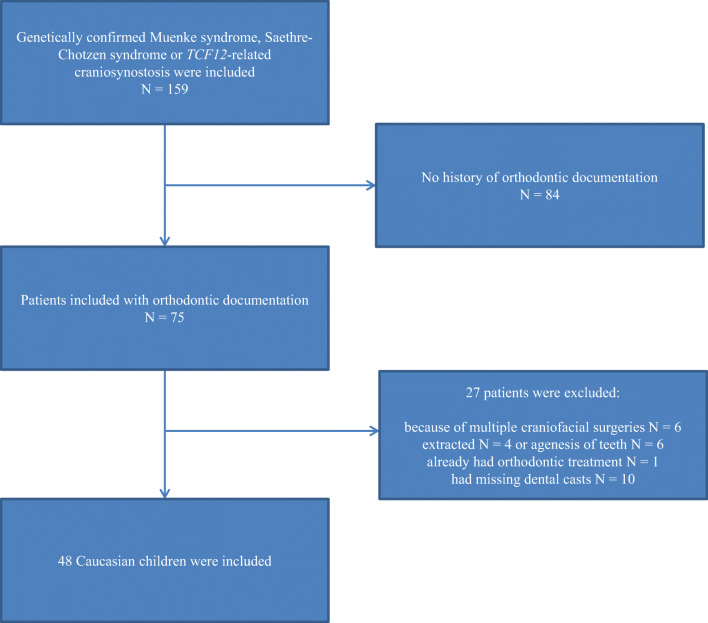
Flowchart describing the inclusion and exclusion criteria of patients and the final study group

The case group comprises of 159 patients that were seen or treated by the craniofacial team between 1990 and 2017 in the Erasmus University Medical Center Rotterdam, the Netherlands. Patients with a Caucasian background and a genetically confirmed Muenke syndrome (P250R mutation in *FGFR3*) (*n* = 82), Saethre-Chotzen syndrome (mutations in or re-arrangements in *TWIST1*) (*n* = 47), or *TCF12*-related craniosynostosis syndrome (mutations or re-arrangements in *TCF12)* (*n* = 30) were included. The clinical diagnosis was made by an expert, i.e., a clinical geneticist or a plastic surgeon. In all patients, this diagnosis was molecularly confirmed by dideoxy sequencing of *FGFR3* (Muenke syndrome), dideoxy sequencing and FISH analysis of *TWIST1* (Saethre-Chotzen syndrome), and dideoxy sequencing of *TCF12* or next-generation sequencing. Medical reports of the patients were screened for a history of orthodontic treatment, tooth extraction treatment, multiple craniofacial surgeries, and available dental casts. The selected dental plaster casts in this study were all taken prior to any orthodontic treatment and any second craniofacial surgery. Reference points for measurements on teeth were clearly identifiable. Eighty-four patients had no history of orthodontic treatment. Six patients had undergone multiple craniofacial surgeries and were therefore excluded (Muenke *n* = 2, Saethre-Chotzen syndrome *n* = 4). Patients with malformed, extracted (*n* = 4) or agenesis of (*n* = 6) teeth were excluded because of possible smaller dental arch dimensions. Ten patients were excluded because of missing dental casts. One patient had finished orthodontic treatment prior to the first visit to our craniofacial team and was therefore excluded. The final sample consisted of 48 Caucasian patients (25 females and 23 males) with a mean age of 8.96 years (SD 2.46) (Fig. 1). Of these, 34 patients had Muenke syndrome, 8 patients had Saethre-Chotzen syndrome, and 6 patients had *TCF12*-related craniosynostosis. The patients of the Saethre-Chotzen syndrome and *TCF12*-related craniosynostosis are grouped because of genetic similarities. Except for one patient, all selected patients in the final sample had undergone a craniofacial surgical procedure according to the treatment protocol of the craniofacial team in the Erasmus University Medical Center Rotterdam.

#### Control group

The data of the control group were obtained from the Nijmegen Growth Study (NGS), which was a population-based cohort study that was conducted from 1971 to 1976 at the University of Nijmegen, the Netherlands. The NGS was a mixed longitudinal, interdisciplinary study of growth and development of healthy Dutch children between 4 and 14 years. Only Caucasian children were included in this study. Examination of the children took place every 6 months.

These data include the repeated measurements of the different arch morphology characteristics of 461 children born between 1961 and 1968. For this case-control study, we selected the arch measurements of the children taken at the age that was closest to the mean age of the Muenke and Saethre-Chotzen syndromes and *TCF12*-related craniosynostosis study group, i.e., 8.96 years. Afterwards, we excluded children that had not all of the arch measurements available (*n* = 132) to avoid the problem of missing values in the analysis.

Thus, in the present study, a healthy control sample of 329 subjects with a mean age of 8.93 years (0.91) was used for the analysis (165 females and 164 males).

Details on the study design and data collection of the NGS sample are explained elsewhere [16].

### Outcome measures

Validated measurements were made on digitally scanned study models [17]. The outcome measures for both jaws in the present study were intercanine width (ICW), intermolar width (IMW), arch depth (AD), and arch length (AL) (Digimodel 2.3.7, OrthoProof B.V, the Netherlands). The same measurements were used in an earlier study on Apert and Crouzon syndrome [4]. The measurements were repeatedly taken on 20 randomly selected dental casts with a 2-week interval in between to investigate intraexaminer reliability. Additionally, a second investigator measured the same 20 dental casts for the assessment of the interexaminer reliability. Ratios for the different dental arch measurements (ICW, IMW, AD, AL) were calculated by dividing the maxillary dental arch measurement by the corresponding mandibular dental arch measurement. The ratio of the maxillary and mandibular arch measurements was used as a proxy for the assessment of intermaxillary relationship.

### Outcome variables

**Fig. 2:**
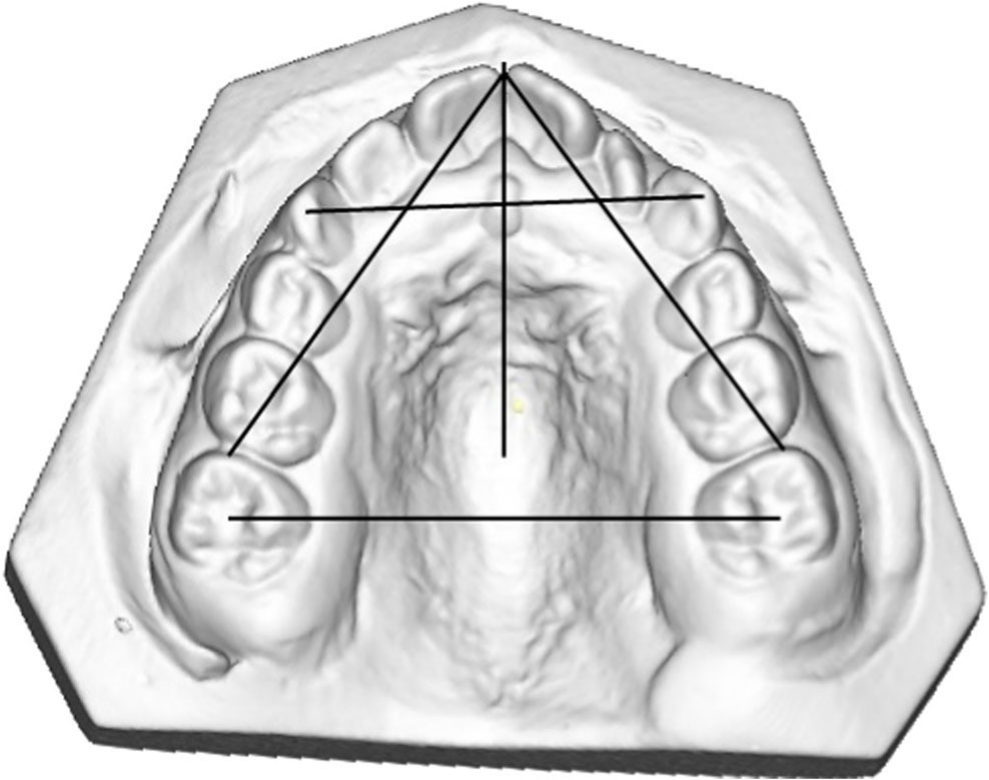
Maxillary digital dental cast of a patient with *TCF12*-related craniosynostosis. The dental arch measurements that are performed are illustrated by the black lines

Eight measurements (Fig. 2) were performed in order to examine the morphology of the dental arches. Four measurements were performed in the maxilla and four in the mandible. One investigator (TC) performed all measurements for further statistical analysis.

(I) The intercanine width was defined as the distance between the cusp tips of the deciduous or permanent canines. In case of cuspal wear, the measuring point was determined as the middle part of the worn cusps.
(II) The intermolar width was defined as the distance between the central point of the occlusal surfaces of the first permanent molars.
(III) The arch depth was defined as the distance measured from the labial surfaces of the central incisors, perpendicular to the line that connects the mesial surfaces of the permanent first molars - if not visible, then the distal surfaces of the premolars or the deciduous second molars were used.
(IV) The arch length was defined as the distance measured from the mesial surfaces of the permanent molars to the interproximal contact point of the central incisors or midpoint of the central diastema. If the permanent molar was not visible, then the distal surface of the permanent second premolar or the second deciduous molar was used [4].

### Statistics

Intraexaminer and interexaminer reliability were calculated with the intraclass correlation coefficient (ICC). A correlation coefficient of 0.75 or higher was considered as a high degree of reliability [18]. We used descriptive statistics to characterize the study population presenting absolute numbers with percentages for categorical data and mean values with standard deviation for continuous data. We compared the sample characteristics of the case group (Muenke separately from the group SCS/*TCF12*-related craniosynostosis) and the control group with the chi-square or ANOVA tests.

The differences in mean values of the different arch dimensions and ratios (ICW, IMW, AD, AL) between the cases, and the controls were compared with linear regression models corrected for age and gender. We build for each measurement a separate model with the case status as independent variable and the arch measurement (ICW, IMW, AD, AL, respectively) as dependent variable. In each model, we added age and gender as covariates to correct for its effect on the effect sizes. We presented the beta (β) and 95% confidence intervals (95% CIs) that are obtained from the models. The β indicates the mean shortening in the arch measurement (mm) of the case group in comparison to the mean arch measurement of the control group independent of eventual age or gender differences. For all analysis, a *p* value of α ≤ 0.05 was considered significant.

All statistical analyses were performed in IBM SPSS Statistics 21 (NY, USA).

## Results

### Intraclass correlation coefficient

The ICC for intrarater reliability ranged from 0.98 to 0.99. The ICC for interexaminer reliability ranged from 0.94 to 0.99.

### Study population

The study population consisted of 34 patients with Muenke syndrome, 8 patients with Saethre-Chotzen syndrome, 6 patients with *TCF12*-related craniosynostosis syndrome, and 329 healthy children.

Of the 34 patients with Muenke syndrome, 18 children were boys and 16 children were girls. Of the 14 patients with Saethre-Chotzen syndrome or *TCF12*-related craniosynostosis, 5 children were boys and 9 children were girls. The mean age of Muenke syndrome and Saethre-Chotzen syndrome or *TCF12*-related craniosynostosis was, respectively, 8.95 years (SD 2.63) and 8.97 years (SD 2.04). Of the 329 healthy children, 164 children were boys and 165 were girls. The mean age of the controls was 8.93 years (SD 0.91). Between the groups, statistically significant differences in gender were found (*p* < 0.000).

Sample characteristics of the study population are presented by gender in Table 1. The size of the study population was 48 members. However, only 37 members could be included in the analysis because of missing values (maxillary: ICW *n* = 11; IMW *n* = 9; AD *n* = 1; AL *n* = 2; mandibular: ICW *n* = 5; IMW *n* = 8).

**Table 1:** Characteristics of the study population

	Controls	Muenke	Saethre-Chotzen syndrome or *TCF12*-related craniosynostosis	*p* value
Age				
Mean ± SD	8.93 ± 0.91	8.95 ± 2.63	8.97 ± 2.04	0.985
Gender				
Boys	164	18	5	
Girls	165	16	9	< 0.000
ICW maxillary				
*n*	329	27	10	
Mean ± SD	31.04 ± 2.19	24.88 ± 3.43	28.31 ± 2.60	< 0.000
ICW mandibular				
*n*	329	31	12	
Mean ± SD	26.12 ± 2.09	23.74 ± 2.42	23.73 ± 2.42	< 0.000
IMW maxillary				
*n*	329	26	13	
Mean ± SD	44.29 ± 2.63	40.40 ± 3.28	41.01 ± 4.00	< 0.000
IMW mandibular				
*n*	329	27	13	
Mean ± SD	40.82 ± 2.61	38.53 ± 2.45	40.86 ± 2.74	< 0.000
AD maxillary				
*n*	329	34	13	
Mean ± SD	28.98 ± 2.22	27.94 ± 2.57	28.17 ± 2.68	0.022
AD mandibular				
*n*	329	34	14	
Mean ± SD	24.29 ± 2.17	23.12 ± 2.07	24.19 ± 1.85	0.011
AL maxillary				
*n*	329	34	12	
Mean ± SD	95.81 ± 6.49	66.61 ± 4.52	67.98 ± 5.88	< 0.000
AL mandibular				
*n*	329	34	14	
Mean ± SD	87.28 ± 6.55	58.80 ± 3.25	61.83 ± 3.62	< 0.000

Continuous data are presented as mean values in mm with standard deviation (mean ± SD); categorical values are presented as absolute numbers and percentages; *p* value is based on one-way ANOVAs for continuous variables and chi-square tests for categorical variables

*ICW* intercanine width, *IMW* intermolar width, *AD* arch depth, *AL* arch length

### Muenke syndrome versus control group

In the children with Muenke syndrome, we found statistically significantly smaller maxillary ICW (β = − 6.06, 95% CI − 6.91, − 5.20), mandibular ICW (β = − 2.43, 95% CI − 3.17, − 1.68), maxillary IMW (β = − 4.83, 95% CI − 5.84, − 3.82), mandibular IMW (β = − 2.83, 95% CI − 3.82, − 1.84), maxillary AD (β = − 1.06, 95% CI − 1.85, − 0.27), mandibular AD (β = − 1.19, 95% CI − 1.95, − 0.43), maxillary AL (β = − 29.33, 95% CI − 31.30, − 27.36), and mandibular AL (β = − 28.60, 95% CI − 30.64, − 26.55) compared to those of the control group (Table 2).

**Table 2:** Differences in arch measurements between patients and control group (β [95%CI])

	Controls	Muenke	Saethre-Chotzen syndrome or *TCF12*-related craniosynostosis
ICW maxillary	Ref	− 6.06^*^	− 1.99^*^
		[− 6,91, − 5.20]	[− 3.37, − .60]
ICW mandibular	Ref	− 2.43^*^	− 2.24^*^
		[− 3.17, − 1.68]	[− 3.41, − 1.07]
IMW maxillary	Ref	− 4.83^*^	− 3.40^*^
		[− 5.84, − 3.82]	[− 4.78, − 2.02]
IMW mandibular	Ref	− 2.83^*^	− 0.07
		[− 3.82, − 1.84]	[− 1.45, 1.31]
AD maxillary	Ref	− 1.06^*^	− 0.80
		[− 1.85, − 0.27]	[− 2.04, 0,44]
AD mandibular	Ref	− 1.19^*^	− 0.02
		[− 1.95, − 0.43]	[− 1.17, 1.12]
AL maxillary	Ref	− 29.33^*^	− 28.66^*^
		[− 31.30, − 27.36]	[− 31.88, − 25.43]
AL mandibular	Ref	− 28.60^*^	− 25.25^*^
		[− 30.64, − 26.55]	[− 28.35, − 22.15]

Data are presented as betas with their 95% confidence interval based on linear regression models adjusted for age and gender. Significant differences are presented with asterisks (p value <0.05)

*ICW* intercanine width, *IMW* intermolar width, *AD* arch depth, *AL* arch length

### Saethre-Chotzen syndrome or *TCF12*-related craniosynostosis patients versus control group

The maxillary and mandibular ICW in the Saethre-Chotzen syndrome or the *TCF12*-related craniosynostosis were statistically significantly smaller than those of the control group (β = − 1.99, 95% CI − 3.37, − 0.60; β = − 2.24, 95% CI − 3.41, − 1.07, respectively). The maxillary IMW in the Saethre-Chotzen syndrome or the *TCF12*-related craniosynostosis was statistically significantly smaller than those of the control group (β = − 3.40, 95% CI − 4.78, − 2.02). The mandibular IMW was not statistically significantly different in the Saethre-Chotzen syndrome or the *TCF12*-related craniosynostosis than those of the control group (β = − 0.07, 95% CI − 1.45, 1.31). The AD in both jaws was not statistically significantly different in the Saethre-Chotzen syndrome or the *TCF12*-related craniosynostosis than those of the control group (maxillary AD: β = − 0.80, 95% CI − 2.04, 0.44; mandibular AD: β = − 0.02, 95% CI − 1.17, 1.12, respectively). The maxillary and mandibular AL in the Saethre-Chotzen syndrome or the *TCF12*-related craniosynostosis was statistically significantly smaller than those of the control group (β = − 28.66, 95% CI − 31.88, − 25.43; β = − 25.25, 95% CI − 28.35, − 22.15, respectively) (Table 2).

### Ratios

The ratios for the ICW and IMW were statistically significantly smaller in Muenke patients than those of the control group (*p* < 0.001 and *p* = 0.016, respectively). The ratio for the AD was not statistically significantly different in Muenke patients than those of the control group (*p* = 0.405). The ratio for the AL was statistically significantly larger in Muenke patients than those of the control group (*p* = 0.013) (Table 3).

**Table 3:** Comparison of maxillary and mandibular ratios between Muenke patients and healthy Dutch children

	Control group		Muenke syndrome		
Measures	*N*	Mean (SD)	*N*	Mean (SD)	*p* value^a^
ICW	329	1.19 (0.08)	25	1.07 (0.11)	< 0.001
IMW	329	1.09 (0.05)	26	1.05 (0.07)	0.016
AD	329	1.20 (0.10)	34	1.21 (0.12)	0.405
AL	329	1.10 (0.05)	34	1.13 (0.08)	0.002

^a^*p* value based on independent *t* test

The ratio for the ICW was not statistically significantly different in the Saethre-Chotzen syndrome or the *TCF12*-related craniosynostosis patients (ICW, *p* = 0.191). The ratio for the IMW was statistically significantly smaller in the Saethre-Chotzen syndrome or the *TCF12*-related craniosynostosis patients than those of the control group (*p* = 0.006). The ratios for the AD and AL were not statistically significantly different in the Saethre-Chotzen syndrome or the *TCF12*-related craniosynostosis patients and those of the control group (AD, *p* = 0.300 and AL, *p* = 0.672, respectively) (Table 4).

**Table 4:** Comparison of maxillary and mandibular ratios in Saethre-Chotzen syndrome or *TCF12*-related craniosynostosis patients versus healthy Dutch children

	Control group		Saethre-Chotzen syndrome or *TCF12*-related craniosynostosis		
Measures	*N*	Mean (SD)	*N*	Mean (SD)	*p* value^a^
ICW	329	1.19 (0.08)	10	1.23 (0.10)	0.191
IMW	329	1.09 (0.05)	12	1.00 (0.08)	0.006
AD	329	1.20 (0.10)	13	1.17 (0.10)	0.300
AL	329	1.10 (0.05)	12	1.09 (0.08)	0.672

^a^*p* value based on independent *t* test

## Discussion

The results of this retrospective case-control study indicate that the dental arch dimensions in Muenke patients were statistically significantly smaller than those of the control group. When we compared the dental arch dimensions of patients with Saethre-Chotzen syndrome or *TCF12*-related craniosynostosis, we found that the dental arch dimensions in Saethre-Chotzen syndrome or *TCF12*-related craniosynostosis patients were also statistically significantly smaller than those of the control group, except for the mandibular IMW and for the AD in both jaws.

To our knowledge, no other study has evaluated the dental arch dimensions in patients with Muenke syndrome and Saethre-Chotzen syndrome or *TCF12*-related craniosynostosis syndrome and has compared those to a healthy control group. As seen in Apert and Crouzon patients, the smaller maxillary transverse dimensions in Muenke and Saethre-Chotzen syndromes or *TCF12*-related craniosynostosis patients may be the result of maxillary constriction and the fusion of the maxillary surrounding sutures [4]. Circumstantial evidence suggests that the fusion of sutures is probably not only concentrated in the cranial vault and at the cranial base but also involves facial sutures and cartilages [4, 19, 20]. As indicated before in Apert and Crouzon patients, the smaller mandibular arch dimensions in Muenke and Saethre-Chotzen syndromes or *TCF12*-related craniosynostosis patients are probably the result of a lingual compensatory growth direction of mandibular teeth towards the narrow maxilla [4, 21].

It is unclear why the mandibular IMW and AD dimensions in Saethre-Chotzen syndrome or *TCF12*-related craniosynostosis patients were not statistically significantly different from those of the control group. We suggest that the maxilla surrounding sutures may be fused at a later stage compared to those of Apert and Crouzon children, thereby allowing a more normal maxillary growth pattern to occur in these patients.

When we compared the ratios in Muenke patients, we found that the ratios of the ICW and the IMW were statistically significantly smaller than those of the control group. Surprisingly, we found that the AL ratio was statistically significantly larger and that the AD ratio was not statistically significantly different in Muenke patients than those of the control group. The normal AD ratio in Muenke patients can be elucidated by a compensatory growth pattern that also occurs in the cranium of craniosynostosis patients. The cranium allows growth in the direction perpendicular to the prematurely fused sutures. In scaphocephaly, for example, compensatory growth occurs in the sagittal direction when the sagittal suture is fused. Therefore, we suggest that the Muenke patients in our study group have a severely fused midpalatal suture resulting in compensatory growth to occur in the maxillary AD. This compensatory growth pattern was not found in Saethre-Chotzen syndrome or *TCF12*-related craniosynostosis patients, which suggest that the maxilla is less constricted than those of Muenke patients. This theory is supported by the ratios for the ICW, AD, and AL in Saethre-Chotzen syndrome or *TCF12*-related craniosynostosis patients, which are not statistically significantly different than those of the control group. We only found that the IMW ratio is statistically significantly smaller. Furthermore, a less restricted maxilla results in a more normal growth pattern that acknowledges the normal ratios in Saethre-Chotzen syndrome or *TCF12*-related craniosynostosis patients.

**Fig. 3:**
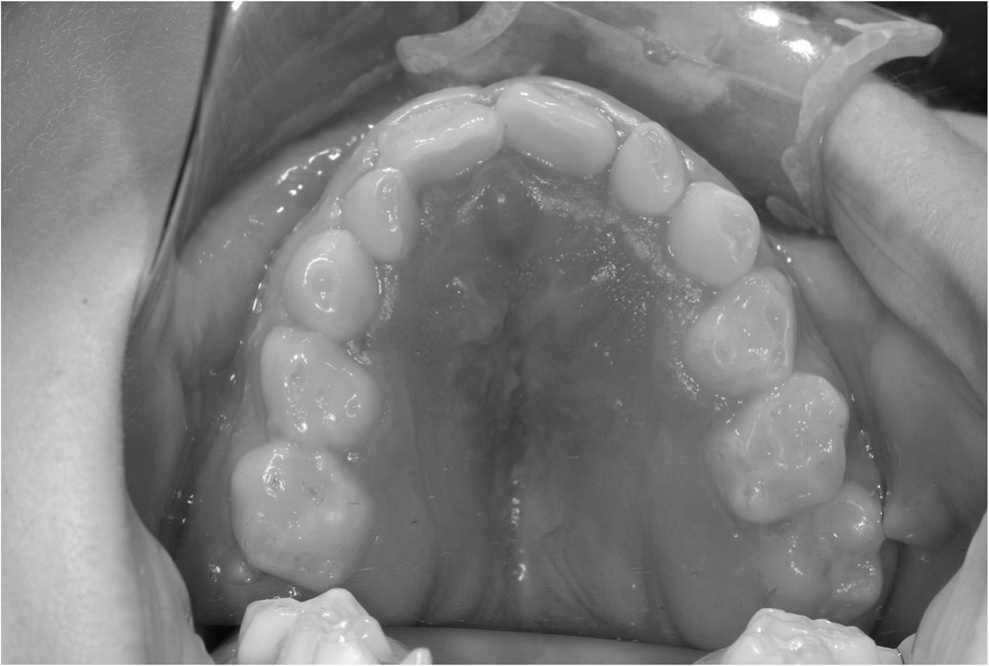
Photograph of a patient with Saethre-Chotzen syndrome. Note the asymmetry of the maxilla on the right side of this 9-year-old male patient with Saethre-Chotzen syndrome

As seen in Apert and Crouzon patients [22], patients with Muenke and Saethre-Chotzen syndromes or *TCF12*-related craniosynostosis are prone to have posterior crossbites [23]. The smaller maxillary transverse dimensions and ratios in our syndromic patients may be related to a specific asymmetric crossbite and an end-to-end bite that comprises from the region of the premolar to the contralateral second incisor. This may be an addition to the clinical identification (Fig. 3).

This study has several limitations. Except for one child, all patients in this study had undergone craniofacial surgery during early childhood that may have affected normal growth of the maxilla. It remains unclear whether the smaller dental arch dimensions in patients with Muenke and Saethre-Chotzen syndromes or *TCF12*-related craniosynostosis is the result of craniofacial surgery or if it is syndrome-related or both. Since all patients undergo surgery at a young age, it is impossible to distinguish this further.

Vucic et al. showed a secular trend in dental development in Dutch children who were born between 1961 and 1994 and beyond. These findings suggest that Dutch children who were born in 2003 achieve on average the same dental maturity about 1.5 years earlier than children who were born 40 years earlier [24]. Additionally, a secular trend was observed in other attributes of Dutch children during the observation period such as mean final height, body mass index, and onset of puberty [25–27]. Therefore, we expect that the dental arch dimensions of the Dutch children will increase in conjunction with the secular trend seen in other attributes.

Because the syndromic patients in our study were born approximately 30 to 40 years later than our control patients, we expect that the discrepancy of the dental arch dimensions between the syndromic and control patients is smaller in our study than when we had used children with a similar year of birth.

To further evaluate the involvement of the maxilla surrounding sutures in relationship to the maxillary dental arch dimensions, we suggest a study with the use of 3D CT analysis. With the use of 3D CT methods, the effect of the amount of fusion of the maxillary surrounding sutures and the effect of the inclination of teeth in relationship to the dental arch can be investigated. Furthermore, future studies should be conducted with a larger case group to increase generalizability by pooling of data and include more covariables to further explore the most often seen asymmetry in patients with Muenke and Saethre-Chotzen syndromes or *TCF12*-related craniosynostosis. In this study, we may have introduced selection bias by the retrospective design of our study. Furthermore, we suspect that we have not included all patients with Muenke and Saethre-Chotzen syndromes or *TCF12*-related craniosynostosis because of their inconspicuous phenotype. Patients with Muenke syndrome and Saethre-Chotzen syndrome or *TCF12*-related craniosynostosis can more easily be overlooked compared with children with Apert and Crouzon syndrome.

The clinical implications for orthodontic treatment of patients with Muenke and Saethre-Chotzen syndromes or *TCF12*-related craniosynostosis lie in the high prevalence of smaller dental arch dimensions and posterior crossbites that should be seen in relation to craniosynostosis. In a case series of CT scans in healthy children that were undergoing rapid palatal expansion, it was shown that the maxillary sutures were expanded and the maxillary molars were inclined [28]. Therefore, orthodontic expansion of the maxilla should be done with care to prevent inclination of molars.

To gain better understanding of the sutural involvement in the midface and support treatment capabilities of medical and dental specialists in these patients, we suggest the concentration of patients with Muenke and Saethre-Chotzen syndromes or *TCF12*-related craniosynostosis in specialized teams for a multidisciplinary approach and treatment.

## Conclusion

In this study, we showed that the maxilla and mandible in Muenke and Saethre-Chotzen syndromes or *TCF12*-related craniosynostosis are smaller in the transverse dimension compared to those of the control group. The analyzed deviant transverse and sagittal ratios indicate that Muenke patients have an abnormal intermaxillary relationship. However, the ratios seen in Saethre-Chotzen syndrome or *TCF12*-related craniosynostosis suggest that these patients have a more normal growth pattern compared to Muenke patients. Additionally, the AD in both jaws of patients with Saethre-Chotzen syndrome or *TCF12*-related craniosynostosis was not statistically significantly smaller compared to those of the control group.
